# Relationships between Physical Activity and Selected Chronic Diseases among Functionally Independent Long-Term Care Residents during the Post-Lockdown Period in Croatia

**DOI:** 10.3390/ijerph20136301

**Published:** 2023-07-05

**Authors:** Ivana Crnković, Karmen Lončarek, Danica Železnik, Sanja Ledinski Fičko, Tomislav Vlahović, Robert Režan, Goran Knežević

**Affiliations:** 1 Department of Physiotherapy, University of Applied Health Sciences, 10 000 Zagreb, Croatia; 2 Department of Ophthalmology, Faculty of Medicine, University of Rijeka, 51 000 Rijeka, Croatia; karmen.loncarek@uniri.hr; 3 Faculty of Health and Social Sciences Slovenj Gradec, 2 380 Slovenj Gradec, Slovenia; 4 Department of Nursing, University of Applied Health Sciences Zagreb, Mlinarska cesta 38, 10 000 Zagreb, Croatia; 5 Clinic for Traumatology, Clinical Hospital Center Sestre Milosrdnice, 10 000 Zagreb, Croatia; 6 Department of Clinical Medicine, University of Applied Health Sciences, 10 000 Zagreb, Croatia; 7 Clinical Hospital Center Zagreb, 10 000 Zagreb, Croatia; 8 Faculty of Humanities and Social Sciences, University of Zagreb, 10 000 Zagreb, Croatia

**Keywords:** geriatrics, retirement home, physical activity, chronic diseases, COVID-19

## Abstract

The aim of this observational study was to investigate the level and association of physical activity and selected chronic diseases in functionally independent LTC residents after prolonged physical and social isolation during COVID-19 in Croatia. Adhering to the inclusion criteria, 180 functionally independent residents were included in the study. Assessment of physical activity was carried out by 7-day motor monitoring. Prolonged physical and social isolation negatively affected the achieved level of physical activity of LTC residents ($\bar{x}$ = 5058.74). Major depressive disorder resulted in significantly lower residents’ physical activity scores, demonstrating a shrinking effect ranging from 0.42 to 0.45. A significant negative impact on the residents’ physical activity was also found in the presence of osteoarthritis and iron deficiency anemia, where a downward effect was present in the range from 0.66–0.72 and 0.64 to 0.66. The presence of comorbidities has a significant negative impact on the residents’ physical activity, where a downward effect is present in the range from 0.91–0.92.

## 1. Introduction

A global public health problem for older adults is chronic diseases, often affected by genetic predispositions, lifestyle and social behaviour, environmental determinants of health, factors of the health care system and the influence of the community [1]. As expected, the prevalence of chronic diseases increases with chronological age, and consequently, the presence of multimorbidity, one of the most complex challenges of clinical and research activity in the 21st century. Based on the latest survey conducted by the SHARE programme in 2020, about 36% of seniors aged 65 and over reported the presence of at least two chronic diseases. As a rule, living with chronic diseases does not result in functional limitations of older adults; however, a third of older adults have at least one limitation in basic and/or instrumental activities of daily life [2,3,4,5]. The presence of chronic diseases, especially the occurrence of multimorbidity, results in the development of frailty, impaired balance, reduced functional capacity, premature institutionalization and more frequent hospitalization with an increased risk of mortality [6,7,8].

Physical inactivity is a significant risk factor in the incidence of chronic diseases and the fourth leading cause of mortality on a global level [9,10]. Also, the presence of chronic diseases, especially in older age groups, significantly contributes to physical inactivity with the practice of prolonged sedentary behaviour, which, in turn, may result in the development of other chronic conditions [11]. However, the literature suggests that the inclusion of older adults with chronic diseases in physical activity is of great public health importance to ensure a high-quality, healthy and long lifespan as well as participation in the social community [12,13]. According to the recommendations of the World Health Organization, multi-component physical activity of moderate intensity for five or more days per week is the most effective measure of preventive health care, but also the most prescribed intervention in geriatric health care, resulting in health benefits even for people with chronic diseases and secondary prevention of other chronic conditions [14,15,16]. The benefits are not only reflected in delaying the onset of chronic diseases, but also in increasing the functional ability and independence of older adults, and in prolonging life. Despite the well-known health benefits of physical activity in this age group, the presence of physical inactivity, and the sedentary lifestyle typical of long-term care (LTC) residents is a pressing problem in geriatric health care [17,18,19].

In the context of the COVID-19 pandemic, older adults with chronic diseases had a higher risk for severe forms of the coronavirus disease; thus, public health restriction measures were maintained for the longest duration in long-term care facilities [20]. In Croatia, strict public health restriction measures came into force in March 2020 following a model which stipulated the limitation of social events and gatherings in the closed spaces of the facility unit, maintaining a minimum distance of 1.5 m in all rooms while wearing a medical mask, banning residents from going outside the long-term care facility unless for medical reasons and a ban on visits by family members and friends. The measures were relaxed in May 2021 [21].

The aim of this study is therefore twofold: (1) to investigate the level of physical activity of functionally independent LTC residents, and (2) to investigate the connection between physical activity and selected chronic diseases after prolonged physical and social isolation during the COVID-19 pandemic in Croatia.

## 2. Materials and Methods

### 2.1. Study Design, Setting, and Participants

The selection of participants in the observational study was carried out from May to October 2021. The study was carried out in facilities for long-term care which are government-owned and have founding rights held by the city of Zagreb. These totaled eleven organizational units.

Croatian long-term care comprise assisted living facilities and provide social accommodation services which include the service of organized living in an apartment or room with personal space hygiene maintenance, use of common rooms and preparation of three meals a day with the application of nutritional norms and monitoring of the nutritional status of the resident. A multidisciplinary gerontological team operates within the facility care and provides services and support in activities of daily life and health care, social work, psychosocial support and rehabilitation, physiotherapy care, occupational therapy activities and counselling, depending on the needs and preferences of the resident. The intensity of the service provided to older adults depends on the type of service, the resident’s needs (i.e., the resident’s functional ability and health status) and is determined by the intensity of the service activities in 1–4 levels [22,23].

Initially, 215 older residents were selected regardless of their gender. The sample size was determined based on the ratio of the total population of people over 65 years of age (N = 154,053) in the city of Zagreb and the target population of LTC residents (N = 3856) in Zagreb, which make up 2.5% of the total observed population [24,25]. The study included participants 65 years of age and older with a diagnosed chronic disease according to the ICD-10 classification [26] lasting at least 5 years, stationed in a long-term care facility within the first level of services in the social welfare system (accommodation), which categorizes the resident as functionally independent—a resident who meets their needs independently without the help of another person. The residents who passed the initial motor testing with 6MWT (Six Minute Walk Test), the TUG test (Timed Up and Go Test) and the FIM (Functional Independence Measure) were included for 7-day motor monitoring. Methodological foundation along with good psychometric characteristics are the reason for including these instruments in the selection of participants [27,28,29].

**Figure 1:**
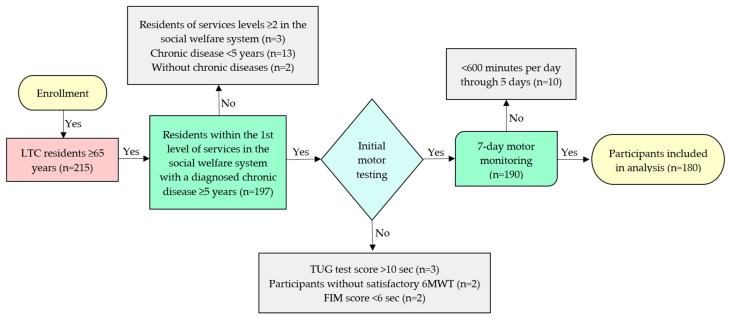
Inclusion and exclusion criteria of the study.

The exclusion criteria for participation in the study were: (1) inability to obtain informed consent drawn up in accordance with the Health Care Act of the Republic of Croatia and the Patients’ Rights Act of the Republic of Croatia [30,31], (2) residents without chronic diseases, (3) diagnosed chronic disease according to the ICD-10 classification lasting less than 5 years, (4) residents of services levels 2–4 in the social welfare system or decompensated patients with psychotic symptoms, moderate and severe types of dementia, severe cognitive impairment, with chronic diseases in exacerbation, with diseases in acute stage with high temperature, with a disorder of consciousness, (5) participants without satisfactory 6MWT, (6) TUG test score above 10 s, (7) FIM score of less than 6 (see Figure 1).

### 2.2. Measurements

For the 7-day motor monitoring, the residents wore a fitness Bluetooth bracelet on their wrists during the 12-h waking period. The fitness Bluetooth bracelet had the following properties: water resistance, small dimensions, simple interface, resistance to microtraumas, and precise recording of output data on the resident’s physical activity. The data tracked by the smart bracelet included the number of steps taken, distance travelled and energy expenditure depending on the resident’s participation in physical activity. Before wear, each participant’s Bluetooth bracelet was connected to an Android device with an activated application for proactive health monitoring, enabling the display and storage of a 12-h record for an individual over a 7-day period. Residents were able to access their daily and weekly statistics after synchronizing with the equipment with prior authorization. At the end of the 7-day motor tracking, all collected data was checked to detect potential issues such as not wearing the fitness Bluetooth bracelet, or incorrectly attaching it; these errors were detected for long periods without any changes in the application. Valid days of wearing the fitness Bluetooth bracelet were considered to be a minimum of 600 min. per day through 5 days to create valid data for the test week. Participants who did not wear the device according to the given parameters were excluded from further processing; a total of 180 participants were included in the data analysis.

### 2.3. Procedure

At the beginning of the project, a multidisciplinary gerontological team, trained to ensure the reliability of the collected data during the planned measurement procedures, was selected within each facility unit. Before beginning data collection, the principals of all facility units accepted participation in the study with formal consent. A physiotherapist and a social worker participated to include residents in the study and in the initial motor testing. Nurses collected relevant health data from the residents’ personal records. All members of the multidisciplinary gerontological team followed the residents daily during the 7-day motor monitoring. The study team applied the Recommendations for preserving the health of people aged 60+ and suffering from chronic diseases. Also applied were protective measures against respiratory infections, including SARS-CoV-2, for people with chronic diseases and older people as advised by the Croatian Institute of Public Health, and Recommendations for preserving the health of people over 65 and the chronically ill (COVID-19) by the Reference Center for Health Care of Older People of the Ministry of Health [32,33,34].

## 3. Data Analysis

Multiple linear regression using a stepwise model was performed for each measure of physical activity as a dependent variable as well as indicators of each confirmed chronic disease, and the total number of confirmed diseases and demographic variables formed predictors. Because the dependent variables were not normally distributed, logarithmic transformation was applied to each dependent variable prior to analysis. Multiple linear regression models were developed for each of the log-transformed measures of physical activity (i.e., three separate models were developed). The predictors were selected using the stepwise model-selection method. All analyses were performed using SAS© Ver. 9.4 (Cary, NC, USA) [35].

## 4. Results

The average age of participants in the study was 81.18 years old (SD = 4.54). The majority of participants included in the survey were female, 73.3%, and males made up 26.7% of the sample. The largest percentage of participants had achieved secondary (73.9%) and higher education (13.9%). The highest percentage of the participants, 60.0%, declared themselves to be widowed, while 21.7% lived in a marital or extramarital unions. The average body height of the participants was 166.2 cm (SD = 9.23), while the average body weight of the participants was 71.36 kg (SD = 9.59). The largest share of participants had two (47.8%) or three or more (31.7%) chronic conditions. According to the ICD-10 categorization of diseases, the largest share of participant disease belonged to the categories of neurological diseases and diseases of the musculoskeletal system and connective tissue. The participant characteristics are summarized in Table 1.

The average indicators of the participants’ achieved level of physical activity according to the results of the 7-day motor monitoring depending on the presence of chronic conditions are shown in Table 2, Table 3, Table 4 and Table 5.

## 5. Multiple Regression

Statistically significant (*p* < 0.05) chronic disease indicators in the prediction of physical activity of participants during the COVID-19 pandemic are shown in Table 6. Regression parameter estimates, exponent of parameter estimates and respective *p*-values are shown in three columns for each of the three models developed (i.e., three columns for each of the three physical activity measures). It should be noted that, because of the logarithmic transformation of the dependent variables, the column titled “exp(Par.Estimate)” is used in the interpretation of the results. Major depressive disorder is a statistically significant predictor of physical activity, demonstrating a shrinking effect ranging from 0.42 to 0.45, corresponding to a decrease of between 58% and 55%. Osteoarthritis and iron deficiency anemia have also proven to be statistically significant predictors of physical activity where a downward effect is present in the range from 0.66 to 0.72 and 0.64 to 0.66. The number of comorbidities was a significant variable in the models where 7-day recordings of average distance traveled in kilometers and calorie consumption were used as dependent variables. It was observed that for an increase in the number of comorbidities by 1, there was an 8% (0.92) lower distance traveled and 9% (0.91) lower calorie consumption, on average.

## 6. Discussion

The results of this study suggest several key findings. According to the results of the 7-day motor monitoring, the functionally independent LTC residents with chronic diseases achieved a low level of physical activity after prolonged physical and social isolation during the COVID-19 pandemic ($\bar{x}$ = 5058.74 steps). The literature suggests that the COVID-19 lockdown period for older adults living in the community and in LTC residents resulted in significant reductions in physical activity accompanied by significant motor and functional declines, and an increase in prolonged sedentary behaviour [36,37,38]. Yamada et al. reported that in initially non-frail older adults in the community during the three waves of the pandemic, the total time of participation in physical activity decreased compared to the period before the pandemic. In older adults living alone and socially inactive, the time spent in physical activity was significantly reduced and these individuals were exposed to a higher risk of adverse incidents [39]. Nygård et al. suggest that the restrictions related to the COVID-19 period negatively affected the level of physical activity in relatively highly educated and active older adults living in the community [40]. Compared to older adults in the community, public health restrictions, regardless of the waves of the pandemic, were not relaxed in long-term care facilities, where restrictions were enforced the longest. Possible reasons for the low involvement of functionally independent persons after the relaxation of public health restriction measures may be a significant loss of muscle strength along with a physical unwillingness to exercise due to insufficient physical activity during the lockdown period [41], as the residents spent most of their time in their personal space. It is possible that the slower process of adaptation to old living conditions in the post-lockdown period resulted in weaker adaption to physical activity, the previous negative attitude towards recreational activities, but also the negative attitude of health and non-health personnel regarding the inclusion of residents in physical activity due to the duration of the still current public health epidemiological measures. The fear of re-infection with the SARS-CoV-2 virus is a potential reason for not implementing the recommended level of physical activity of LTC residents in the post-lockdown period, due to the re-establishment of contact with other residents, most often owing to the discrepancy between the residents’ needs and the requirement to maintain social distance, as well as the environments in which gerontological activity takes place, such as narrow corridors and the inadequate adaptation of the dimensions of exercise rooms, which would allow a distance between the residents of at least 1.5 m, but also the inadequate adaptation of the facilities, depending on the number of LTC residents [42]. It is necessary to highlight that the study was carried out at a time when public health restrictions were eased and when the residents had the opportunity to participate in certain recreational activities in the external spaces of the facility unit while observing the given epidemiological measures. It is also important to point out that the use of the mHealth technology to maintain health status, such as regular exercise, provides a better potential for resistance to the pandemic. Since all sports and recreational activities in the facility unit were halted during the lockdown period, the residents not using the mHealth technology could have a significant negative impact on the achieved level of physical activity in the post-lockdown period [43].

Results of the study suggest that the presence of major depressive disorder in the COVID-19 pandemic correlates with residents’ physical activity (*p* < 0.05). Major depressive disorder or clinical depression is a complex disorder and one of the most common in terms of prevalence; in the COVID-19 pandemic it significantly affected older adults. It is diagnosed when a person has a constant bad and depressed mood, reduced interest in pleasant activities, feelings of guilt or worthlessness, lack of energy, reduced concentration, changes in appetite, psychomotor restlessness or agitation, sleep disorders or suicidal thoughts [44,45]. Lebrasseur et al. report that the restrictive measures during the lockdown period resulted in a reduced quality of life, but also in increased concerns about health, poorer sleep quality and an increase in depressive symptomatology in this age group [46]. Docherty et al. suggest that perceived stress, well-being, mood disorders, memory and depressive symptomatology significantly worsened in older adults compared to the pre-pandemic period. The follow-up results suggested that well-being, mood, and depressive symptomatology continued to have a negative impact in the post-lockdown period [47]. Studies suggest that the COVID-19 pandemic has had a significant negative impact on the mental health of older adults who live in the community as well. Briggs et al. reported increased depressive symptomatology in older adults who live in the community, especially in those over 70 and/or those living alone [48]. Sepúlveda-Loyola et al. suggests that social distancing during the COVID-19 has resulted in increased depression, anxiety, poor sleep quality and physical inactivity among older adults who live in the community [49]. Cortés Zamora et al. in a cohort longitudinal study report that after a three-month follow-up of residents without moderate or severe cognitive impairment in long-term care facilities, clinically significant symptoms of depression were recorded regardless of the COVID-19 status [50]. We find a multidimensional association between physical inactivity and prolonged sedentary behaviour, and an increase in depressive symptomatology in LTC residents during the COVID-19 pandemic. Studies suggest that older age is a risk factor for the incidence of depression, and LTC residents are a particularly vulnerable group [51]. Compared to other age groups, older adults spend the most time in sedentary activities, approximately 80% of their waking time between 8 and 12 h a day, which has a significant negative impact on the presence of depression [52]. Regardless of the reasons for institutionalization, we know that it represents an additional challenge for older adults. Facility units have the ability to provide health and social care with high technical conditions and professional staff, but research shows that older adults experience a sudden motor decline and impairment of the functional status after entering a facility unit [53]. The majority of older adults prefer living in a family community despite the need for health and social care, therefore institutionalization can potentially have a negative impact on the prevalence of depression in this group [54]. Public health restriction measures resulted in a change in personal habits of LTC residents with a decrease in social interaction with other residents within the facility unit, which contributed to an increase in perceived social isolation that tends to result in a faster decline in cognitive abilities with weaker individual functioning as well as an increased risk of developing depressive disorders. At the same time, banning residents from leaving the premises of the facility unit resulted in a decrease in physical activity and an increase in sedentary behaviour, which is a significant risk factor for an increase in depressive symptoms, especially in the older age [55,56,57]. On the other hand, we find several studies that suggest that practicing mentally active sedentary behaviour, such as board games, in order to socialize and communicate with friends, can have a protective effect on the occurrence of depression, in contrast to mentally passive sedentary behaviour such as watching television [58]. During the current epidemiological measures, all social activities in long-term care facilities were halted, and residents spent most of their time alone in their personal space and practiced prolonged sedentary activities such as watching television, which could potentially have a significant impact on the results in the post- during the lockdown period. Compared to older adults in the community, LTC residents are not inclined to use social networks in order to communicate and socialize with family members and friends, and during the lockdown period their communication in this segment was limited, which possibly contributed to the significant negative impact of depressive symptomatology on the achieved participant physical activity [59].

Osteoarthritis and iron deficiency anemia also proved to be statistically significant predictors of physical activity in LTC residents (*p* < 0.05). Osteoarthritis and iron deficiency anemia are some of the leading health, social and economic problems, often resulting in worsening of pain control and intensity during activity, increased generalized fatigue, increased physical inactivity, worsening of motor and functional deficits with the development of secondary chronic conditions [60,61]. Cegla and Magner state that patients with chronic pain in the COVID-19 pandemic period experienced changes in the biopsychosocial area which affected their general well-being. They reported that increase in pain was related to changes caused by the pandemic, but also the noticeable deterioration in pain control [62]. Endstrasser et al. report in a prospective study that VAS pain scores and WOMAC questionnaire results increased significantly during the lockdown period, while the physical activity significantly decreased. In the final evaluation, VAS and WOMAC showed a significant negative correlation with the Tegner Activity Scale [63]. The worsening of pain in the COVID-19 period in older adults with osteoarthritis is suggested by the results of the study done by Román Belmonte et al. [64]. Clinically, older adults with osteoarthritis report the presence of pain and fatigue as significant limiting factors in the domain of physical activity [65,66]. Studies suggest an association of iron deficiency anemia with reduced muscle mass and strength, reduced motor ability and the presence of disability. Also, people with anemia more often practice a sedentary lifestyle and are physically less active compared to others because of the increased presence of fatigue [67,68]. The studies suggest a multidimensional interaction of pain symptoms and fatigue in the domain of physical activity [69,70]. The presence of pain can result in fatigue and vice versa, and the presence of pain and fatigue results in a partial or complete cessation of physical activity. Physical inactivity over a longer period of time leads to postural imbalance with a decrease in motor and functional ability, which at the same time leads to a deterioration of control and an increase in the intensity of pain and fatigue. Studies suggest that the prolonged lockdown period had an additional effect on the increase in sedentariness [71], especially in people who previously practiced a sedentary lifestyle, which possibly resulted in increased pain and fatigue precisely in these diseases, and secondarily in additional inactivity in LTC residents.

The occurrence of comorbidities results in worse health outcomes, requires complex clinical management and results in increased health care costs, and the pandemic period had devastating consequences for people with comorbidities, especially in the older age group [72,73]. In their study, Ismail et al. report that during the pandemic there was a significant worsening of the symptoms of existing chronic diseases, and Delpino et al. emphasize that in a group of 516 people, as many as 27.1% developed multimorbidity in the period from the 1st to the 2nd pandemic wave [74,75]. Ruzafa-Martinez et al. report that older adults with multimorbidity suffered a significant deterioration in functional and cognitive abilities during the pandemic period [76]. The health characteristics and physical behavior of the participants, but also the different duration and models of implementation of the public health restriction measures in each country, resulted in the commitment of researchers in the pandemic period depending on the achieved level of physical activity and the presence of chronic diseases. In their meta-analysis, Pérez-Gisbert et al. report that the pandemic period had a significant negative impact on the level of physical activity achieved by people with chronic diseases, which resulted from isolation, restriction of social activities and social distancing [77]. On the other hand, Esain et al. report in their study that older adults who were involved in an exercise program before the pandemic remained physically active during the pandemic, regardless of their functional status [78]. The association of physical inactivity after prolonged COVID-19 restriction measures and multimorbidity in an older adult is multidimensional and should be viewed through heterogeneity in the clinical and functional statuses. It is positive that the results of this study suggest that the presence of comorbidities has a lower impact on the achieved level of physical activity of residents compared to individual chronic diseases such as depression, osteoarthritis and iron deficiency anemia. It should be emphasized that the participants in this study were involved in a social welfare programme and counselling work with physiotherapy treatment, which could potentially have a significant impact on the achieved results of physical activity. High-functioning residents were included in this study, and we can assume that the initial motor, functional and nutritional status could have a significant impact on physical activity. Although this topic is the subject of future research, these results may suggest that the occurrence of comorbidities is not of crucial importance in achieving the recommended physical activity level.

## 7. Suggestions for Future Research

As an observational study only association but not causation can be inferred. Previous studies suggest that the use of fitness bracelets can have a significant impact on the achieved level of physical activity, which may have influenced the results in this study, and in subsequent studies it is necessary to use a greater number of methods of assessing physical activity. 

## 8. Conclusions

According to the results of this study, prolonged physical and social isolation during the COVID-19 lockdown had a negative effect on the achieved level of physical activity in functionally independent LTC residents. Major depressive disorder resulted in significantly lower residents’ physical activity scores. A significant negative impact on the resident’s physical activity is also found in the presence of osteoarthritis and iron deficiency anemia. The presence of comorbidities has a significant negative association on the residents’ physical activity. The results of this study may facilitate the creation of guidelines for preventive public health programs in geriatric health care with the aim of increasing the physical activity of LTC residents.

## Figures and Tables

**Table 1:** Baseline characteristics of participants.

Demographic Characteristics			
Age ($\bar{x}$ = 81.18, SD = 4.54)			
		**N**	**%**
Gender	M	48	26.7
	F	132	73.3
Marital status	Unmarried	12	6.7
	Lives in a marital or extramarital union	39	21.7
	Divorced	21	11.7
	Widowed	108	60.0
Educational level	No education	6	3.3
	Primary school	16	8.9
	High school	133	73.9
	Bachelor’s degree	11	6.1
	Master’s degree	14	7.8
**Anthropometric characteristic**			
Height ($\bar{x}$ = 166.2, SD = 9.23)			
Weight ($\bar{x}$ = 71.36, SD = 9.59)			
**Health characteristics**			
		**N**	**%**
Number of chronic diseases	1	37	20.6
	2	86	47.8
	≥3	57	31.7
Comorbidities	Angina pectoris	7	3.9
	Asthma	6	3.3
	Cerebrovascular accident	27	15.0
	Type 2 Diabetes Mellitus	28	15.6
	Hypertension	115	63.9
	Chronic Obstructive Pulmonary Disease (COPD)	7	3.9
	Chronic bronchitis	9	5.0
	Osteoarthritis	42	23.3
	Osteoporosis	25	13.9
	Major depressive disorder, recurrent	6	3.3
	Suffered from heart attack	17	9.4
	Rheumatoid arthritis	56	31.1
	Iron deficiency anemia	18	10.0
	Cardiac arrhythmia	20	11.1

**Table 2:** Physical activity level (all participants).

	$\bar{x}$	SD	Minimum	Maximum	Median
**Steps**	5058.74	3237.66	482	17,567	4441.00
**Distance** (km)	3.22	2.08	0.33	10.71	2.67
**Energy Expenditure** (kcal)	114.36	85.04	8.00	442.00	93.50

**Table 3:** Physical activity level (the presence of a single chronic disease).

	$\bar{x}$	SD	Minimum	Maximum	Median
**Steps**	5940.73	3527.10	1361.00	16,631.00	5661.00
**Distance** (km)	3.82	2.26	0.96	9.64	3.37
**Energy Expenditure** (kcal)	139.97	94.23	24.00	382.00	110.00

**Table 4:** Physical activity level (the presence of two chronic diseases).

	$\bar{x}$	SD	Minimum	Maximum	Median
**Steps**	5660.28	3412.77	482.00	17,567.00	4921.50
**Distance** (km)	3.59	2.18	0.33	10.71	2.97
**Energy Expenditure** (kcal)	128.17	89.35	9.00	442.00	101.00

**Table 5:** Physical activity level (the presence of three or more chronic diseases).

	$\bar{x}$	SD	Minimum	Maximum	Median
**Steps**	3578.63	2128.58	484.00	9945.00	3173.00
**Distance** (km)	2.29	1.41	0.34	6.99	2.00
**Energy Expenditure** (kcal)	76.89	55.98	8.00	269.00	66.00

**Table 6:** Multiple linear regression results (three models for three measures of physical activity).

	Log (Steps)			Log (Distance)			Log (Energy Expenditure)		
	Par. Estimate	Pr > F	exp(Par. Estimate)	Par. Estimate	Pr > F	exp(Par. Estimate)	Par. Estimate	Pr > F	exp(Par. Estimate)
Major depressive disorder	−0.86	0.0001	0.42	−0.80	0.0002	0.45	−0.83	0.0008	0.44
Osteoarthritis	−0.36	0.0001	0.70	−0.33	0.0004	0.72	−0.42	0.0001	0.66
Iron deficiency anemia	−0.44	0.0010	0.64	−0.41	0.0024	0.66	−0.43	0.0073	0.65
Comorbidities	-	-	-	−0.08	0.1360	0.92	−0.10	0.1270	0.91
Age	−0.05	<.0001	0.95	−0.05	<.0001	0.95	−0.07	<.0001	0.94
Educational level	0.15	0.0038	1.16	0.15	0.0050	1.16	0.16	0.0105	1.17
Male participants	0.28	0.0020	1.32	0.28	0.0018	1.32	0.35	0.0007	1.43

Note: COPD had to be eliminated from multiple linear regression because of linear dependency among predictors (when COPD was included).

## Data Availability

The data presented in this study are available upon request from the corresponding author.
